# Early life Conditions and Trends in Mortality at Later Life: Is There any Relationship?

**Published:** 2011

**Authors:** Masoud Amiri

**Affiliations:** 1 Department of Epidemiology and Biostatistics, Medical Plants Research Center, Shahrekord University of Medical Sciences, Shahrekrod, Iran. Email:m.amiri@skums.ac.ir

Mortality from ischemic heart disease (IHD),^1^ stroke,^2^,^3^ and gastric cancer^4^,^5^ have fallen during recent decades, especially in developed countries; however, IHD and stroke remain among the top leading causes of death in Europe.^6^,^7^ Furthermore, wide geographic variations in mortality rates and trends from these diseases have been shown since the early 1950s^8^,^9^ and for incidence and case fatality of IHD by the WHO MONICA Project at the mid-1980s.^10^

There is a controversy exist in the relationship between early life conditions and mortality at later decades of life. Several studies have shown the association between IHD and inappropriate living conditions in early lifetime,^11^–^15^ but not always.^16^,^17^ Some studies have observed this relationship for stroke,^14^,^18^ whereas others have not.^12^,^15^ Time trends of gastric cancer also differ between populations and the effect of living conditions on trends is not yet fully understood.^19^

In this paper, the results of my previous population-based time-series studies would be discussed that aimed to describe mortality trends between subsequent cohorts considering living conditions in early decades of life of these cohorts.^20^,^21^ Cohort-wise trends of IHD, stroke, and gastric cancer mortality of populations in seven European countries -i.e., Denmark, England and Wales, Finland, France, the Netherlands, Norway and Sweden- have been assessed to determine whether these trends were correlated with developments of infant mortality rate (IMR) of the subsequent cohorts.

Generally, an overall decline in mortality rate from these three diseases from 1950 to 2005 in all countries, in all age ranges and both men and women have been observed, with the important exception of a period epidemic of IHD at 1950 to 1970. In a perspective cohort, we observed steady declines for between cohorts performed between 1860 and 1939 in most causes, except for IHD.

Mortality due to IHD increased between the 1950s and 1970s in all countries for both men and women, except for younger women in France and Finland.^20^ This increase was generally followed by a decline between the 1970s and 1990s, particularly among younger people. In most countries, the decline started earlier among women than men. However, in all countries, the decline was slightly more in male than female, in particular in the Netherlands, Sweden and Denmark. The overall decline persisted until 2005, with no consistent evidence for a deceleration.

Mortality from stroke decreased from 1950s to 1990s in all age ranges, and both sexes, in most countries, often with larger declines in the recent decades.^20^ The rate of these declines greatly differ according to birth cohort, country and sex, with mostly higher declines in the youngest birth cohorts, suggesting cohort effects related to possibly high smoking epidemic. Relatively strong declines were observed in France, compared to smaller declines in Sweden. The observed declines persisted until 2005.

For stroke, when data of all countries were pooled, and associations across countries were also taken into account, the overall correlation between IMR and mortality rate was observed to be strong. This strong association with stroke mortality rate may suggest the relation between early life conditions and adult hypertension, that is rather strong^22^ and which in turn may contribute to associations with stroke mortality. In separate analyses in which we made comparisons across countries, instead of birth cohorts, the relationship was slightly positive comparing countries for birth cohorts born before 1900. However, for birth cohorts born after 1900 this association was slightly negative. This inconsistency in patterns will be discussed below.

The relationship of IMR and IHD mortality seem to be more complex. A variable and sometimes inverse relationship was observed in different countries. After pooling all countries, and also taking the associations across countries into account, the overall correlation with IMR was not significant for IHD mortality. Clearly the ‘epidemics’ of IHD - of which the rise and fall still are ill-understood^16^,^17^ - do not parallel the ending of the ‘epidemics’ of IMR. The cohort-wise decline in IHD mortality rate showed a hill-like shape, which would disturb the relation with the universally declining IMR. After an initial decline, a temporary increase was present which, together with the subsequent decline, can be described as the rise and fall of the IHD epidemic in general. The causes of this epidemic are not entirely understood. The increasing smoking epidemic is an insufficient explanation, if only because the smoking epidemic affected men and women in different ways, whereas the timing of the IHD epidemic was similar for both sexes. Some have pointed the potential role of adaptation to an entire different life and nutrition pattern during the first half of the 20^th^ century.^23^,^24^

In all countries, gastric cancer mortality rates declined steadily for all birth cohorts, all ages, and both sexes.^21^ The smallest decrease was belonged to Norwegian men and the largest decline to Danish and Finnish women. Especially in the 1980s, the Finnish mortality rate declined to attain levels as in other countries. The rate of decline remained similar over time in most other countries. The decline in gastric cancer over successive cohorts was generally stronger among women than men. For both sexes, the observed declines persisted until 2005. A strong association was found between gastric cancer and IMR across different cohorts in all countries. When the different countries were pooled, the overall correlation with gastric cancer mortality was positive for IMR at the time of birth.

Despite the fact that many of the relations between the three diseases and IMR were as expected, some irregularities were observed, in particular for IHD and gastric cancer.

In addition, period-wise changes in medical care may have confounded the study of the associations between IHD mortality trends and early life factors. These associations may have been obscured amongst others by large changes in effective medical care during the second half of the 20^th^ century.^23^,^24^ In stroke and gastric cancer, change in care may have been more gradual, although the invention and introduction of life saving therapies (ambulance transport, CCU, thrombolytics, etc) may also have introduced large period-wise changes in some of the countries studied.

Even though IMR and stroke were strongly related, the irregularities in the decline of IMR did not clearly correspond to similar irregularities in the decline of stroke. Stroke mortality rates were relatively stable in birth cohorts from 1860 to 1880, and declined in subsequent cohorts, but not as rapidly as IMR. We can only speculate about why the general IMR improvement did not translate as quickly into a cohort-wise decline in stroke mortality. It could be due to that late IMR improvement is based on factors with broader health effects (with lasting effects also on stroke incidence and mortality) compared the factors most important earlier in IMR improvement (e.g. reduction of exposure through new sewerage systems). Or, the elderly population in general changes over time, with a more heterogeneous group entering old age in later cohorts, which in turn may change the time relation between initial IMR and stroke mortality in later birth cohorts.

We believe that the discrepancies that we observed between IMR trends and the ones for IHD and stroke mortality warns against too strong statements regarding an overriding effect of early life conditions on population-wide trends in these diseases. In addition, my studies suggested the importance of “environment” in early decades of life in determining the risk of gastric cancer,^7^,^25^ particularly childhood social circumstances,^26^–^28^ which is consistent with the strong geographical correlation between mortality from gastric cancer in adulthood and infant mortality.^17^,^29^ However, some of our results indicate that secular trends in gastric cancer were not neatly related to trends in IMR, suggesting that mortality trends were partly affected by changes in adult socioeconomic circumstances and lifestyles, rather than childhood.
